# PENTAGON (Predicting Clinical Trials in Gynecologic Oncology): A Retrospective Study Assessing Study Design Factors That Affect Enrollment in Gynecologic Cancer Trials

**DOI:** 10.3390/cancers17233774

**Published:** 2025-11-26

**Authors:** Margaret Klein, Humza Pirzadah, Yasmeen Magharehabed, LaMiah Hall, Andrew Chapple, Navya Nair, Tara Castellano, Amelia Jernigan

**Affiliations:** 1 Rush University Medical Center, Chicago, IL 60612, USA; 2 New Orleans School of Medicine, Louisiana State University, New Orleans, LA 70112, USA; 3 Division of Biostatistics, School of Public Health, Louisiana State University Health Sciences Center, New Orleans, LA 70112, USA; 4 Division of Gynecologic Oncology, University of Miami Sylvester Comprehensive Cancer Center, Miami, FL 33136, USA; 5 Division Gynecologic Oncology, Louisiana State University Health Sciences Center, New Orleans, LA 70112, USA; 6 University Medical Center New Orleans, LCMC Health, New Orleans, LA 70112, USA

**Keywords:** gynecologic oncology, cancer clinical trials, retrospective cohort study, enrollment barriers

## Abstract

*Objective*: Clinical trials are the cornerstone of medical innovation and set the standard for medical care. Cancer clinical trials drive critical innovation and availability of new treatments. However, obstacles to successful recruitment and participation of patients in clinical trials still significantly limit their success. Our objective is to evaluate trial enrollment trends according to both patient and trial demographics/features for those who have sought care at our multi-center, mixed-setting Gynecologic Oncology practice, allowing for us to describe specific trial criteria designs that are negatively associated with diverse enrollment or lack of enrollment completion on cancer clinical trials. *Methods*: An IRB-approved, retrospective cohort study was completed to evaluate patients who screened positive for a clinical trial through the Gynecologic Oncology practice’s manual screening process. We looked at how trial characteristics affect enrollment success. *Results*: After adjustment, there were no significant differences between patient factors and enrollment status. Higher rates of enrollment were seen among Asian patients and patients whose race was reported as “unknown” (>50%, *p* = 0.125). There was a drastic, unadjusted estimated increase in enrollment for Medicaid patients compared to patients with other insurance types (55.2% vs. 32%, *p* = 0.031), but multivariable logistic regression showed that insurance status (Medicaid vs. others) lost significance, *p* = 0.148. Patients with Stage I cancer accounted for 42.1% of patients enrolled, and enrollment rates were higher than for other cancer stages (*p* = 0.087). There was a significant increase in enrollment likelihood if the trial did not exclude subjects with prior cancer (50.0% vs. 33.3%, *p* = 0.046). *Conclusions*: We demonstrated equitable trial enrollment across different races and insurance statuses and were able to identify criteria that lend itself towards higher rates of enrollment. These findings can be used to tailor cancer trial portfolios to a diverse patient catchment.

## Simple Summary

Clinical trials are a crucial part of healthcare because they can be used to find new and advanced treatments for patients. However, trials are significantly limited due to the lack of enrollment by participants, especially those of diverse backgrounds. In our paper, we analyzed gynecologic cancer trials, and factors that increased or limited patient participation. We analyzed factors, such as insurance type, race, and cancer stage, and how they might affect whether a patient successfully enrolled in a trial. We found that patients with Medicaid insurance and those with Stage I cancer were more likely to enroll. Race and insurance type also played a role, but some differences were not significant after adjusting for other factors. Additionally, trials that did not exclude patients with a history of prior cancer had higher enrollment rates. Our study signifies that certain factors can enhance enrollment and lead to clinical trial success. This finding can be helpful for researchers in the future, because they can model clinical trials to include factors that maximize participation.

## 1. Introduction

Clinical cancer trials establish the standard of care in a field that is marked by constant innovation and novel therapeutics. Clinical trial participation has also been shown to directly benefit participants’ overall health and healthcare outcomes. In a 2017 meta-analysis by Nijjar et al. evaluating 21 studies, it was shown that trial participation significantly improved the survival rate of women with ovarian cancer compared to non-participants [1]. Cultivating a tailored and robust clinical trial menu to maximize enrollment has the potential to improve outcomes for individual patients and the broader population and should be a priority in cancer care.

However, the success of clinical trials is limited by recruitment and participation of patients. Over 80% of clinical trials do not enroll enough patients, and roughly 19% of trials end due to insufficient enrollment [2]. Of the identified barriers to trial participation, the majority disproportionately impact minority and underserved populations. Although the 1993 Revitalization Act of the National Institutes of Health adopted a policy mandating the inclusion of minorities and women, there is still an underrepresentation of various ethnic and racial minorities, women, individuals of lower socioeconomic status, elderly, and rural patient populations in clinical trials [1,3,4,5,6,7,8,9,10]. To promote trial enrollment, it is critical that we understand our patient population, their barriers and preferences, and what makes a trial appealing to those that are eligible [7,11,12,13,14,15,16,17,18,19,20,21,22,23,24,25,26].

At our medium-sized, mixed academic–community Gynecologic Oncology practice, we offer a wide variety of gynecologic cancer clinical trials: cancer care delivery and therapeutic trials, phase I–IV. Our gynecologic oncologists administer chemotherapy and so have continuity with patients throughout the cancer journey. We serve a diverse, mixed urban–rural catchment. We have had success bolstering trial enrollment to over 20% of our gynecologic cancer patients, with intentional emphasis on trials and care delivery that would be appealing to those in our catchment that are typically marginalized (such as black women and rural patients). However, there has been no formal evaluation of which specific patient or trial factors impact eventual trial enrollment versus enrollment failure. We felt it would be beneficial to plainly describe specific trial design elements that are associated with trial enrollment. Our objective is to evaluate trial enrollment trends according to both patient and trial demographics/features for those who have sought gynecologic cancer care with our team.

## 2. Methods

An IRB-approved, retrospective cohort study was completed to evaluate all patients who screened positive for a clinical trial through the Gynecologic Oncology practice’s manual screening process. Screening events were defined as: (1) new neoplasms, (2) new cancer diagnosis for established patients, (3) recurrences or progressions of known disease, (4) changes in patient’s treatment regimen, or (5) discussion of a patient’s case at bi-monthly Gyn Onc tumor boards. Definitions of these events are detailed in Table 1. Statistical analyses were then performed as described below.

Data was collected from July 2022 to December 2023. This included demographic information for each patient, trials for which each patient was considered, trials patients were enrolled on, and each trial’s individual characteristics, including disease site, funding source, biopsy requirements, and specific inclusion and exclusion criteria. The timeline was also considered, including when new trials were opened, so that the heterogeneity of trial characteristics was fully appreciated. A list of all trials that were available at our practice and descriptions of each trial are presented in Supplementary Table S1.

Patient data was obtained retrospectively from the electronic medical records of the LSU Health Gynecologic Oncology Division, encompassing the University Medical Center New Orleans (UMCNO), East Jefferson General Hospital (EJGH), and West Jefferson Medical Center (WJMC) sites. Data were collected using a standardized abstraction form approved under the institutional IRB protocol, which outlined the primary data sources, extraction process, and verification steps. Study personnel recorded demographics, cancer characteristics, treatment history, and clinical trial enrollment data. All entries were verified for accuracy by cross-referencing source documentation, and a random 10% of records underwent secondary review by the principal investigator for quality control. Data was de-identified prior to analysis, with linkage files stored separately on a secure, password-protected LSU Health network drive in accordance with IRB-approved procedures.

## 3. Statistical Analysis

Categorical variables were summarized using counts and percentages, while continuous variables were summarized as means with standard deviations. Group comparisons were conducted using Fisher’s exact tests for categorical variables and Wilcoxon rank-sum tests for continuous variables. Multivariable logistic regression was performed with enrollment status as the dependent variable. For patient-level analyses, covariates included age, race, ethnicity, insurance status, marital status, cancer stage, and distance from the trial site. For trial-level analyses, covariates included prior cancer history and prior treatment requirements, with adjustment for patient-level factors such as distance, insurance, race, and marital status. Trial-level variables (e.g., prior cancer exclusion, prior treatment requirement) were evaluated separately using logistic regression models adjusted for patient-level covariates such as distance from site, insurance status, race, and marital status. Given the exploratory intent of the study and the modest sample size, variable selection procedures were not performed. All analyses were conducted using R (version 4.3.1; R Foundation for Statistical Computing, Vienna, Austria).

## 4. Results

**Figure 1:**
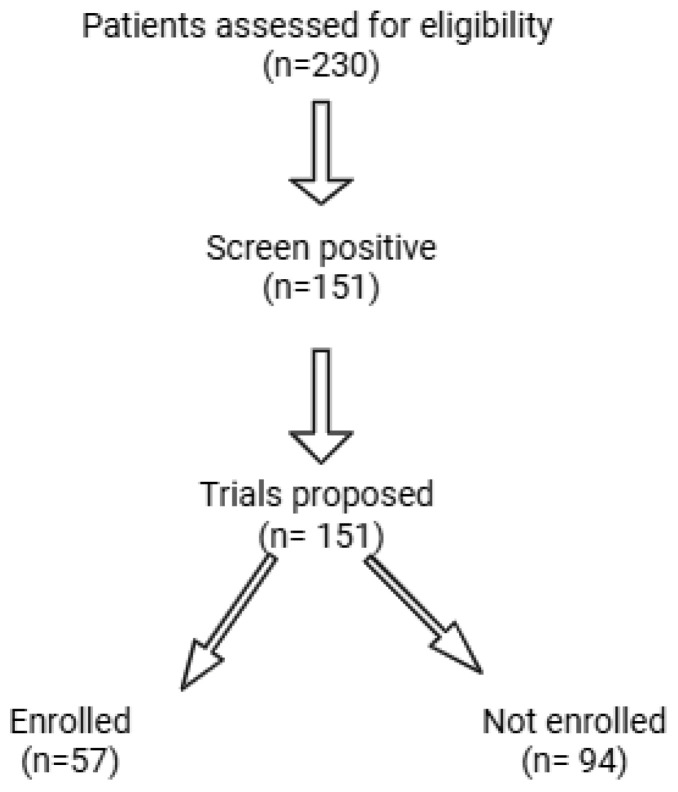
Flow diagram of patient screening and enrollment process. Of 230 patients assessed for eligibility, 151 screened positive for at least one trial, and 57 were enrolled.

A total of 151 patients were included in the final analysis. The flow of patients through screening and enrollment is shown in Figure 1. Of 230 patients assessed for eligibility, 151 screened positive for at least one trial, and 57 of these were ultimately enrolled. The average age of participants was 60.7 years old, and most patients studied presented with endometrial cancer (61.6%). There were a total of 72 Black patients (47.7%) and 63 White patients (41.7%) included, with respective enrollment rates of 31.9% and 34.9%. The rest of the demographic information is presented in Table 2.

The inclusion and exclusion criteria requirements, trial stage, cancer type, and other characteristics of available trials were considered and reported on. In our portfolio, there were eight trials open for endometrial cancer (42.1%), five for cervical cancer (26.3%), and six for ovarian cancer (31.6%). Of these, n = 11 trials required a biopsy (57.9%), and n = 18 required in-person follow-up visits (94.7%). Most studies were interventional based (78.9%), as opposed to observational. Also, there are more therapeutic drug trials (73.7%), as opposed to other interventional/surgical trials. These data are presented in Table 3. The enrollment rates of each individual trial and enrollment rates based on trial characteristics were calculated, as shown in Supplementary Tables S2 and S3. These supplementary tables provide expanded breakdowns of individual trial characteristics and enrollment rates for completeness. When looking at trial characteristics compared to enrollment rates, there was a significant increase in enrollment likelihood if the trial did not exclude subjects with prior cancer (50.0% vs. 33.3%, *p* = 0.046). Clinical trials that required patients to have no prior chemotherapy demonstrated a trend for less successful enrollment compared to those trials which permitted prior chemotherapy 27.3% vs. 44.1%, (*p* = 0.07).

**Figure 2:**
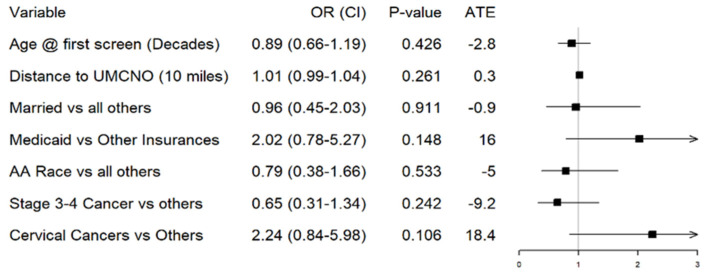
Multivariable logistic regression for whether a patient enrolled in a trial based on demographic and cancer characteristics.

After adjustment, there were no significant differences between patient factors (age, marital status, race, ethnicity, and number of screening events) and enrollment status (Figure 2, Table S4). Enrollment status was defined as a dichotomous variable, with yes/no answer options. The “yes” category indicated that patients were screened, found to be candidates (“screen positive”), and subsequently enrolled on trial. The “no” category included all patients who screened positive at one of the screening events identified by the Gyn Onc team (detailed in Table 1) but ultimately did not enroll on trial. It did not include patients who screened negative, only those who were classified as “screen positive, enrollment failure” events.

There was a drastic, unadjusted estimated increase in enrollment for Medicaid patients compared to patients with private insurance, Medicare, and free care (55.2% vs. 32%, *p* = 0.031). However, a multivariable logistic regression evaluating patient enrollment based on demographic and cancer characteristics showed that insurance status (Medicaid vs. others) lost significance, *p* = 0.148. Marital status was also analyzed across the clinical trials but there was no significant difference for those who enrolled compared to those who did not enroll (*p* = 0.288). There were higher rates of enrollment seen among Asian patients and patients whose race was reported as “unknown” (>50%, *p* = 0.125). These groups also had smaller sample sizes (n = 16) compared to White/Black patients. The aforementioned multivariable logistic regression calculated a *p*-value of 0.533 for the variable of Black race vs. all others. When considering the primary gynecologic cancer sites of patients enrolled in clinical trials, there was no statistically significant association with enrollment and cervical cancer as the primary disease site (15.2% of patients enrolled, *p* = 0.106), compared to other primary disease sites. In our population, we found that patients with Stage I cancer accounted for 42.1% of patients enrolled, which trended higher than the enrollment rates for other cancer stages, though the trend was not found to be statistically significant (*p* = 0.087). Trials that these patients were able to enroll in included SISTER, NRG CC010, FIERCE, NERG GY006, and NRG GY026, which are further described in Supplementary Table S1.

There was also a significant relationship between the distance traveled to the clinical trial site and clinical trial enrollment. Patients who lived within 20 miles of the clinical trial site, enrolled 30.1% of the time (n = 93), compared to 46.6% for those who lived more than 20 miles away (n = 58) (*p* = 0.032). The overall logistic regression model was statistically significant (likelihood-ratio test *p* = 0.004) with a Nagelkerke R^2^ ≈ 0.21, indicating moderate model fit. Although several univariate associations were significant (e.g., Medicaid insurance status, *p* = 0.031; distance from site, *p* = 0.032), no individual covariate retained significance after multivariable adjustment.

## 5. Discussion

Unlike prior reports, we aimed to look at not just how patient characteristics impact clinical trial enrollment, but also how the actual trial characteristics, such as inclusion and exclusion criteria, trial design, or funding source affect enrollment success. Our findings showed equitable trial enrollment across different races and insurance statuses. This suggests that following not only patient demographics, but also trial criteria, is important for tailoring a targeted portfolio to a specific patient catchment.

Furthermore, we showed robust enrollment for Medicaid patients and patients with cervical cancer. While this may reflect our unique patient population, these two characteristics are likely confounding. There is a well-documented higher incidence of cervical cancer in patients who are underinsured, likely due to limited preventative care and screening [27]. This population is often considered hard to engage, especially due to the barriers to care these patients disproportionately face. However, this study shows that these patients, if offered the necessary resources, will participate in clinical research. We were able to identify ways to fill care gaps by evaluating trial demographics and comparing them directly to patient characteristics and will help guide future trial openings to continue to endorse equity.

As an example, we found that patients were more likely to enroll if they lived farther from the site of clinical trials. This is initially counterintuitive because distance is classically thought of as a barrier to care. While it is true that our robust trial portfolio likely attracts participants willing to travel for advanced care. However, in a less resourced population, such as is often seen with cervical cancer and Medicaid, it is crucial to highlight the work we have put into designing a system that helps to overcome these barriers. We have active clinical trial navigation, specifically with virtual options such as a virtual remote nursing program, which provides more opportunities for follow-ups closer to home. We have mechanisms in place to facilitate virtual trial enrollment and follow up activities for patients at remote sites. This has been particularly useful for some of our earlier stage endometrial cancer trials like NRG CC010 and SISTER. Collaboration with community clinics and offering innovative solutions to the barrier created by distance has made this practice a large referral site for trial enrollment, enhancing and expanding our patient catchment area.

Regarding patient presentation, in evaluating our “screen no trial available” population we identified a gap in trial availability for early-stage disease, and we quickly addressed this by opening trials that would include these early-stage frontline patients, like NRG CC010, SISTER, FIERCE, and ROCC. Our center has developed a robust manual clinical trial screening process, which has allowed for efficient trials opening to meet current patient needs [28]. Subsequently, many patients we enrolled in had Stage 1 disease. Of available trials, 42.1% of them targeted primary disease presentation. Patients who had more advanced disease stages and comorbidities are often less likely to participate in clinical trials compared to their counterparts with earlier stage disease [2]. Experimental therapeutics for patients with advanced disease with few treatment options are important to explore. However, this patient population comes with comorbidities and exhaustion from the burdens of what they have been through. Furthermore, early phase investigational therapeutic trials are often high-stakes, costly for both the patient and the institution, and more exclusive to those in excellent health. It remains an important priority, but if we focus exclusively on advanced and end-stages of disease space we risk addressing the particular needs in our specific cancer population served. Trials targeting patients who are earlier in their cancer journeys and for early-stage disease are often focused on complete remission and symptom management. These trials answer critical questions and often expose patients with fewer comorbidities and less disease burden to trials in a lower-stakes environment. Our patient population has shown that they are interested in and willing to enroll in trials in the earliest stages of cancer care. Many describe the experience as rewarding and, when need be, go on to enroll again at disease progression or recurrence.

Trials that excluded patients with a history of chemotherapy had lower rates of enrollment. This is concerning in Louisiana, where we have a “cancer alley” along the corridor from New Orleans to Baton Rouge. To be inclusive of our catchment, we realized early on that we had to be careful about opening trials that would permit patients with prior cancer and cancer therapies to join. As cancer prevalence and survivorship increases so does the likelihood of having had cancer. Accommodating patients with a history of cancer and cancer therapy will be a critical consideration in our goal to be inclusive and applicable to the real world.

Within the confines of improving our clinical trial profile, we wanted to understand both our patient population and how our portfolio is serving that population. This work helps delineate clinical trial criteria that can usher in future enrollment success. Additionally, this study analyzes features of clinical trials but also features of the enrollees. It accounts for social factors that can impact enrollment such as marital status and distance to trial site. Our study has focused on enrollment in gynecologic oncology clinical trials. Approximately 84,000 new cases are diagnosed and about 28,000 deaths occur each year from gynecologic cancer among women in the United States [29]. Thus, it is crucial to investigate factors that can maximize patient enrollment for an area that affects a big population. Furthermore, this study did not exclude clinical trials by date. As many clinical trials as possible were included, maximizing the pool of data to extract from and learn from.

When considering limitations of this study, it is important to recognize that data for the clinical trials included were only collected from a single site, though the trials are cooperative group trials open at multiple sites across the country. Thus, for this “single institution” study, the findings may not be applicable to patient enrollment trends at other institutions or regions of the country. Additionally, due to the pilot nature of this study, we did not have previous data on the most predictive clinical trial characteristics, and how to best classify and track these criteria. Furthermore, the limited trial portfolio decreases the diversity of trials open at the time of our data analysis. This study could be enhanced by collecting patient feedback to enhance understanding of what barriers are deemed most important when considering clinical trial participation. Patient feedback can broaden our understanding of these trends and allow us to further refine clinical trials to maximize patient participation and satisfaction.

The importance of identifying trial design features and patient demographics that significantly impact enrollment can help guide researchers and physicians in developing their trial menus to maximize appeal and likelihood of patient participation in the future. Thus, clinical trials should be adjusted to meet patient needs because patients will readily engage if it is made accessible to them. After this evaluation, we have shown what patient and trial factors had a positive and/or negative impact on enrollment. When going through clinical trial feasibility questionnaires collecting this type of data will be an important resource for cancer sites and selecting the projects that best fit their clinical population and trial team structures.

## 6. Conclusions

In this study, we examined patient and trial level factors that impact patient participation. We found that patients with early-stage disease and those enrolled in trials with more inclusive eligibility criteria had the highest participation rates. In contrast, we observed that excluding patients with a prior history of cancer or chemotherapy limited enrollment, signifying the need for trial designs that reflect real world populations. We also noted higher participation rates among patients willing to travel farther for clinical trials, suggesting that structural support, such as a virtual navigator, plays an important role in overcoming traditional barriers. Future efforts should elaborate on these observations in a larger, multi-institutional context and engage patient perspectives to understand strategies that maximize enrollment and ensure that clinical trials remain accessible, inclusive, and meaningful for the diverse population of gynecologic oncology patients we serve.

## 7. Limitations

We acknowledge that this study has several limitations. First, the number of enrollment events in the logistic regression models was modest, which causes the possibility of overfitting. We limited the analysis to a small set of clinically meaningful variables, but results should be interpreted as exploratory and hypothesis-generating. Second, although non-parametric tests were applied in some comparisons, continuous variables were summarized as means with standard deviations for clinical interpretability, which may not fully capture non-normal distributions. Third, beyond insurance status, additional socioeconomic variables (e.g., income or payment method) were not consistently available and could not be analyzed. Future multi-institutional studies with larger sample sizes and richer sociodemographic data are needed to confirm and expand these results.

This study was presented in part at the Society of Gynecologic Oncology 2024 Meeting by LaMiah Hall, MPH.

## Figures and Tables

**Table 1:** Definition of screening events.

Screening Event	Description
New neoplasm	A patient who presents to the clinic for the first time.
New cancer diagnosis	A patient who is diagnosed with a new cancer. This could be a new patient or an established patient with a new formal diagnosis of cancer.
Recurrence	A patient who has previously been seen by the Gynecologic Oncology team, has received treatment, and was deemed to be in remission. However, there is new evidence that the cancer has returned.
Progression	A patient who has previously been seen by the Gynecologic Oncology team, has received treatment, and is now demonstrating evidence of tumor spread or growth.
Discussion at Tumor Board	The Gynecologic Oncology team hosts bi-monthly tumor boards to discuss difficult cases, trial eligibility, and plans of action for specific patients. It is a collaborative multidisciplinary event where there is joint decision making. Patients discussed at these meetings were screened for trial enrollment.
Change in treatment regimen	If a patient’s treatment regimen changes for any reason (for example—no response to treatment, progression, recurrence, or intolerance) the patient is assessed for enrollment on clinical trial.

**Table 2:** Baseline Patient Characteristics.

Characteristics		N (%)	Characteristics		N (%)
**Age (mean)**		60.7 (13.5)	**Cancer Category**		
**Race**				Endometrial	93 (61.6)
	American Indian/Alaska native	0 (0)		Cervical	23 (15.2)
	Asian	4 (2.6)		Ovarian	28 (18.5)
	Native Hawaiian or Pacific Islander	0 (0)		Vulvar	2 (1.3)
	Black or African American	72 (47.7)		Vaginal	2 (1.3)
	White	63 (41.7)		Uterine	0 (0)
	More than one		**Marital Status**		
	Unknown/Not Reported	12 (7.9)		Divorced	14 (9.3)
**Insurance Status**				Domestic partner	0 (0)
	Private	40 (26.5)		Legally separated	1 (0.7)
	Medicare	72 (47.7)		Married	58 (38.4)
	Medicaid	29 (19.2)		Single	61 (40.4)
	Free care	4 (2.6)		Unknown	3 (2)
	Other	7 (4.6)		Widowed	14 (9.3)
**Distance from UMCNO (mean)**		59.3 (137.4)	**Charlson Comorbidity Index (mean)**		5.9 (2.9)

**Table 3:** Clinical trial characteristics.

Clinical Trial Characteristics		N%
**Clinical Trial Cancer Type**		
	Endometrial Cancer	8 (42.1)
	Cervical Cancer	5 (26.3)
	Ovarian Cancer	6 (31.6)
	Vaginal Cancer	1 (5.3)
	Not Applicable	3 (15.8)
**Biopsy Required**		11 (57.9)
**In Person**		18 (94.7)
**Virtual**		1 (5.3)
**Biomarker**		5 (26.3)
	HER2	2 (10.5)
	BRCA1	1 (5.3)
	FOLR1	1 (5.3)
**Interventional**		15 (78.9)
**Observational**		2 (10.5)
**Surgical**		3 (15.8)
**Immunotherapy Chemotherapy**		11 (57.9)
**Specified Age Range**		1 (5.3)
**Primary Cancer**		8 (42.1)
**Maintenance Phase**		1 (5.3)
**Recurrent or Progressive Cancer**		9 (47.4)
**Pharma Trial**		14 (73.7)
**Non-Pharma Trial**		5 (26.3)
**Trial Design**		
	Parallel Assignment	15 (78.9)
	Single Group Assignment	2 (10.5)
	Crossover Assignment	1 (5.3)
	Sequential Assignment	1 (5.3)
**Systemic** **Treatment Needed Prior to Trial**		
	Chemotherapy/Immunotherapy	2 (10.5)
	Surgery	3 (15.8)
	No Treatment	16 (84.2)
**Phase**		
	1	2 (10.5)
	2	6 (31.6)
	3	7 (36.8)
	Not Applicable	3 (15.8)
**Inclusion Criteria**		
	Race/Ethnicity	2 (10.5)
	No standard therapy available, or standard therapy has failed	2 (10.5)
	Patients must have received no prior therapy including hormonal therapy, chemotherapy, targeted therapy, immunotherapy or radiation therapy	12 (63.2)
	None of the above	6 (31.6)
**Exclusion Criteria**		
	Incarceration	1 (5.3)
	Pregnancy	11 (57.9)
	Unable to consent in English	2 (10.5)
	Evidence of metastatic disease	14 (73.7)
	Comorbidities	16 (84.2)
	Hypertension	6 (31.6)
	Autoimmune Disorders	6 (31.6)
	CDK	2 (10.5)
	Heart Disease	10 (52.6)
	Lung Disease	8 (42.1)
	Liver Disease	6 (31.6)
	Psychiatric Illness	3 (15.8)
	Immunodeficiencies	10 (52.6)
	Previous history of other cancer	12 (63.2)
	Concomitant medications	5 (26.3)
	Currently on another chemotherapy regimen	5 (26.3)
	Previous chemotherapy	5 (26.3)
	Previous surgery	4 (21.1)

## Data Availability

De-identified data supporting the findings of this study are available from the corresponding author upon reasonable request.

## Supplementary Materials

The following supporting information can be downloaded at: https://www.mdpi.com/article/10.3390/cancers17233774/s1, Table S1. Open trials at our Gynecologic Oncology practice. Table S2. Enrollment Rates for Clinical Trials. Table S3. Enrollment Rate Based off Trial Designs. Table S4. Multivariable logistic regression for whether patient factors influence trial enrollment overall.

## Abbreviations

UMCNO	University Medical Center New Orleans, a primary academic affiliate of Louisiana State University (LSU) Health Sciences Center and a participating clinical site for gynecologic oncology trials.
EJGH	East Jefferson General Hospital, an affiliated community site contributing to patient recruitment and trial participation.
WJMC	West Jefferson Medical Center, an affiliated partner institution supporting clinical trial screening and data collection.
LSU	Louisiana State University Health Sciences Center, the coordinating academic institution for study oversight, IRB approval, and research infrastructure.
SISTER	Social Interventions for Support during Treatment for Endometrial Cancer and Recurrence; a multi-site randomized controlled trial evaluating the impact of virtual social and behavioral interventions on survivorship outcomes in endometrial cancer.
NRG CC010	A Phase III Trial of the Impact of Sentinel Lymph Node Mapping on Patient-Reported Lower Extremity Limb Dysfunction in Endometrial Cancer; an NRG Oncology trial assessing functional outcomes following sentinel lymph node procedures.
NRG GY006	Incorporation of Triapine (T) with Cisplatin Chemoradiation (CRT) for Locally Advanced Cervical and Vaginal Cancer; an NRG Oncology Phase III study investigating enhanced chemoradiation strategies for cervical malignancies.
NRG GY026	A Phase II/III Study of Paclitaxel/Carboplatin Alone or Combined with Either Trastuzumab and Hyaluronidase-Oysk (Herceptin Hylecta) or Pertuzumab, Trastuzumab, and Hyaluronidase-Zzxf (Phesgo) in HER2-Positive, Stage I–IV Endometrial Serous Carcinoma or Carcinosarcoma; an NRG Oncology trial evaluating HER2-targeted therapies.
FIERCE	Facilitating Inclusive Enrollment in Research for Cancer Equity; a Phase Ib trial evaluating pembrolizumab com-bined with vaginal cuff brachytherapy and chemotherapy for high-intermediate–risk endometrial cancer.
ROCC	Robotic versus Open Radical Hysterectomy for Cervical Cancer (GOG-3043); a randomized controlled trial comparing surgical approaches to radical hysterectomy in cervical cancer.
