# Supplementary material for: PENTAGON (Predicting Clinical Trials in Gynecologic Oncology): A Retrospective Study Assessing Study Design Factors That Affect Enrollment in Gynecologic Cancer Trials
Source: Cancers (Basel). 2025 Nov 26;17(23):3774. doi: 10.3390/cancers17233774 (PMC12691471; doi:10.3390/cancers17233774)
Supplement: Supplementary file 1 [file cancers-17-03774-s001.zip › cancers-3923940-supplementary.pdf]

cancers

Supplementary Materials

Table S1. Open trials at our Gynecologic Oncology practice.

Copyright: © 2025 by the authors. Licensee MDPI, Basel, Switzerland. This article is an open access article distributed under the terms and conditions of the Creative Commons Attribution (CC BY) license (https://creativecommons.org/licenses/by/4.0/).

Trial Name	Trial Description	Trial Characteristics
SISTER	Social Interventions for Support during Treatment for Endometrial Cancer and Recurrence (SISTER): a multi-site randomized controlled trial	-Endometrial Cancer<br>-Virtual, Interventional, Non-Pharma Trial<br>-Inclusion cx: Race, no prior therapy<br>-Exclusion cx: Incarceration, non-English speaking, Psychiatric Illness
NRG-G019	A Randomized Phase III, Two-Arm Trial of Paclitaxel/Carboplatin/Maintenance Letrozole Versus Letrozole Monotherapy in Patients with Stage II-IV, Primary Low-Grade Serous Carcinoma of the Ovary or Peritoneum	-Ovarian Cancer<br>-In-person, biopsy needed, immuno/chemotherapy, interventional, primary cancer, Pharma trial<br>-Inclusion cx: no prior therapy<br>-Exclusion cx: metastatic disease, currently on other chemo regimen, previous chemotherapy, heart disease, immunodeficiency
Dragonfly	A Phase I/II, First-In-Human, Multi-Part, Open-Label, Multiple-Ascending Dose Study to Investigate the Safety, Tolerability, Pharmacokinetics, Biological, and Clinical Activity of DF1001 in Patients With Locally Advanced or Metastatic Solid Tumors, and Expansion in Selected Indications	-In-person, biopsy needed, specific biomarker (HER2), immuno/chemotherapy, interventional, Pharma trial<br>-Inclusion cx: No standard therapy available or standard treatment has failed, patients have received no prior therapy<br>-Exclusion cx: Pregnancy, metastatic disease, previous history of other cancer, concomitant medications, HTN, autoimmune disease, heart disease, or immunodeficiency
NRG-GY018	Testing the Addition of the Immunotherapy Drug Pembrolizumab to the Usual Chemotherapy Treatment (Paclitaxel and Carboplatin) in Stage III-IV or Recurrent Endometrial Cancer	-Endometrial Cancer<br>-In-person, interventional, recurrent or progressive cancer, Pharma trial<br>-Inclusion cx: No prior therapy<br>-Exclusion cx: Pregnancy, metastatic disease, concomitant medications, autoimmune disease, heart, lung, or liver disease, immunodeficiency
NRG-GY020	A Phase III Randomized Trial of Radiation +/- Pembrolizumab (MK-3475) for Newly Diagnosed, Early Stage High Intermediate Risk Mismatch Repair Deficient (dMMR) Endometrioid Endometrial Cancer	-Endometrial Cancer<br>-In-person, interventional, recurrent or progressive cancer, Pharma trial<br>-Inclusion cx: No prior therapy<br>-Exclusion cx: Pregnancy, metastatic disease, concomitant medications, autoimmune disease, heart, lung, or liver disease, immunodeficiency

CHEMO ID	Standard Chemotherapy versus Cancer Stem Cell Assay Directed Chemotherapy in Recurrent Platinum Resistant Ovarian Cancer	-Ovarian cancer<br>-In-person, biopsy needed, immuno/chemotherapy, interventional, recurrent or progressive cancer, Pharma trial<br>-Exclusion cx: Pregnancy, specific comorbidities, concomitant medications
DIRECT	Disparities in REsults of Immune Checkpoint Inhibitor Treatment (DiRECT): A Prospective Cohort Study of Cancer Survivors Treated with anti-PD-1/antiPD-L1 Immunotherapy in a Community Oncology Setting	-In-person, biopsy needed, immuno/chemotherapy, observational, primary cancer, recurrent or progressive cancer, Pharma trial<br>-Inclusion cx: Race/ethnicity, no prior therapy<br>-Exclusion cx: Previous history of other cancer, currently on other chemotherapy regimen
GOG-3043 ROCC	A Randomized Controlled Trial of Robotic versus Open Radical Hysterectomy for Cervical Cancer	-Cervical cancer<br>-In-person, surgical, interventional, primary cancer, non-Pharma trial<br>-Exclusion cx: Pregnancy, metastatic disease, previous history of other cancer, autoimmune disease
NRG CC010	A Phase III Trial Of The Impact Of Sentinel Lymph Node Mapping On Patient Reported Lower Extremity Limb Dysfunction In Endometrial Cancer	-Endometrial cancer<br>-In-person, biopsy needed, surgical, interventional, primary cancer, non-Pharma trial<br>-Exclusion cx: unable to consent in English, metastatic disease, previous history of other cancer, previous chemotherapy, HTN, comorbidities
NRG GY026	A Phase II/III Study of Paclitaxel/Carboplatin Alone or Combined with Either Trastuzumab and Hyaluronidase-oysk (HERCEPTIN HYLECTA) or Pertuzumab, Trastuzumab, and Hyaluronidase-zzxf (PHESGO) in HER2 Positive, Stage I-IV Endometrial Serous Carcinoma or Carcinosarcoma	-Endometrial cancer<br>-In-person, specific biomarker (HER2), immuno/chemotherapy, interventional, primary cancer, Pharma trial<br>-Inclusion cx: Patients have received no prior therapy<br>-Exclusion cx: Pregnancy, metastatic disease, previous history of other cancer, HTN, heart or lung disease
NRG CC008	A Non-Randomized Prospective Clinical Trial Comparing the Non-Inferiority of Salpingectomy to Salpingo-oophorectomy to Reduce the Risk of Ovarian Cancer Among BRCA1 Carriers [SOROCk]	-Ovarian cancer<br>-In-person, specific biomarker (BRCA1), surgical, specified age range, non-Pharma trial<br>-Exclusion cx: Previous history of other cancer
FIERCE	A Phase Ib trial of vaginal cuff brachytherapy + pembrolizumab (MK3475) followed by 3 cycles of dose dense paclitaxel/q 21 day carboplatin + pembrolizumab (MK3475) in High Intermediate Risk endometrial cancer	-Endometrial cancer<br>-In-person, biopsy needed, immuno/chemotherapy, interventional, primary cancer, Pharma trial<br>-Inclusion cx: Patients have received no prior therapy<br>-Exclusion cx: Metastatic disease, previous history of other cancer, lung or liver disease, psychiatric illness, immunodeficiency
IOVANCE	A Phase 2, Multicenter Study to Evaluate the Efficacy and Safety Using Autologous Tumor Infiltrating Lymphocytes (LN-145) in Patients with	-Cervical cancer

	Recurrent, Metastatic, or Persistent Cervical Carcinoma	-In-person, immuno/chemotherapy, interventional, recurrent or progressive cancer, Pharma trial<br>-Inclusion cx: Patients have received no prior therapy<br>-Exclusion cx: Pregnancy, metastatic disease, previous history of other cancer, autoimmune disease, CKD, heart or lung disease, or immunodeficiency
GLORIOSA	Randomized, multicenter, open-label, phase 3 study of mirvetuximab soravtansine in combination with bevacizumab versus bevacizumab alone as maintenance therapy for patients with FRα-positive recurrent platinum-sensitive epithelial ovarian, fallopian tube, or primary peritoneal cancers who have not progressed after second line platinum-based chemotherapy plus bevacizumab	-Ovarian Cancer<br>-In-person, biopsy needed, specific biomarker, immunotherapy, interventional, maintenance phase, recurrent or progressive cancer, Pharma trial<br>-Inclusion cx: No standard therapy available or standard therapy has failed<br>-Exclusion cx: Pregnancy, metastatic disease, other cancer, currently on other chemotherapy or previous chemotherapy, hx of HTN, heart lung or liver disease, immunodeficiency
ACRIVON	A phase 1b/2 basket study of acr-368 as monotherapy and in combination with gemcitabine in adult subjects with platinum-resistant ovarian carcinoma, endometrial adenocarcinoma, and urothelial carcinoma based on acrivon oncosignature® status	-Endometrial and Ovarian cancer<br>-In-person, biopsy needed, interventional, Pharma trial<br>-Inclusion cx: Patients have received no prior therapy<br>-Exclusion cx: Metastatic disease, HTN, heart or liver disease, or immunodeficiency
STAR	Phase II Trial of Niraparib in Combination with Dostarlimab in Patients with Recurrent or Progressive Cervix Cancer (STAR)	-Cervical cancer<br>-In-person, biopsy needed, immuno/chemotherapy, interventional, recurrent or progressive cancer, Pharma trial<br>-Inclusion cx: Patients have received no prior therapy<br>-Exclusion cx: Pregnancy, metastatic disease, previous history of other cancer, concomitant medications, currently on other chemo, previous chemotherapy or surgery, autoimmune disease, lung or liver disease, or immunodeficiency
A151804	Establishment of a National Biorepository to Advance Studies of Immune-Related Adverse Events	-In-person, biopsy needed, specific biomarker, immuno/chemotherapy, observational, primary cancer, non-Pharma trial<br>-Inclusion cx: Patients have received no prior therapy
Advenchen	Phase 1/2a/3 Evaluation of Adding AL3818 to Standard Platinum-Based Chemotherapy in Subjects With Recurrent or Metastatic Endometrial, Ovarian, Fallopian, Primary Peritoneal or Cervical Carcinoma	-Endometrial, Cervical, and Ovarian Cancer<br>-In-person, biopsy needed, immuno/chemotherapy, interventional, recurrent or progressive cancer, Pharma trial<br>-Exclusion cx: Pregnancy, metastatic disease, previous history of other cancer, previous chemo or surgery, concomitant

		medication, HTN, heart disease, immunotherapy
NRG GY006	Incorporation of triapine (T) with cisplatin chemoradiation (CRT) for locally advanced cervical and vaginal cancer	-Cervical or Vaginal cancer<br>-In-person, immuno/chemotherapy, interventional, primary cancer, recurrent or progressive cancer, Pharma trial<br>-Exclusion cx: Pregnancy, metastatic disease, previous history of other cancer, currently on other chemo, previous surgery, HTN, CKD, heart or lung disease, psychiatric illness

Table S2. Enrollment Rates for Clinical Trials.

Enrollment Rate By Trial	Number of patients Screened Positive	Number of patients enrolled	Number of patients screened and not enrolled	% of Enrollment
A151804	1	1	0	100
ACRIVON	4	2	2	50
Advenchen	2	0	2	0
CHEMO ID	13	6	7	46.2
DIRECT	35	12	23	34.3
Dragonfly	4	1	3	25
FIERCE	6	4	2	66.7
GLORIOSA	5	0	5	0
IOVANCE	1	0	1	0
NRG CC008	1	0	1	0
NRG CC010	31	9	22	29
NRG GY006	1	0	1	0
NRG GY018	4	2	2	50
NRG GY019	5	3	2	60
NRG GY020	2	2	0	100
NRG GY026	4	1	3	25
ROCC	11	7	4	63.6
SISTER	31	14	17	45.2
STAR	1	0	1	0

Table S3. Enrollment Rate Based off Trial Designs.

Enrollment Rate Based on Trial Designs		Number of patients Screened Positive*	Number of patients Screened positive* and enrolled	Number of patients screened and not enrolled	% of Enrollment
Clinical Trial Cancer Type					
	Endometrial Cancer	101	41	60	40.6
	Cervical Cancer	24	11	13	45.8
	Ovarian Cancer	34	11	23	32.4
	Vulvar Cancer	1	0	1	0
	Vaginal Cancer	1	1	0	100
Biopsy Required		107	38	69	35.5
In Person		131	50	81	38.2
Virtual		31	14	17	45.2
Biomarker		15	3	12	20

	HER2	8	2	6	25
	BRCA1	1	0	1	0
	FOLR1	5	0	5	0
**Interventional**		112	45	67	40.2
**Observational**		36	13	23	36.1
**Immunotherapy Chemotherapy**		65	22	43	33.8
**Surgical**		43	16	27	37.2
**Specified Age Range**		1	0	1	0
**Primary Cancer**		94	37	57	39.4
**Maintenance Phase**		5	0	5	0
**Recurrent or Progressive Cancer**		64	22	42	34.4
**Pharma Trial**		87	33	54	37.9
**Non-Pharma Trial**		75	31	44	41.3
**Trial Design**					
	Crossover Assignment	1	0	1	0
	Parallel Assignment	140	52	88	37.1
	Sequential Assignment	4	1	3	25
	Single group assignment	17	11	6	64.7
**Systemic Treatment Needed Prior to Trial**					
	Chemotherapy/ Immunotherapy	15	6	9	40
	Surgery	13	7	6	53.8
	No Treatment	138	51	87	37
**Frequency of follow-up (weeks)**					
	0	11	7	4	63.6
	1	41	19	22	46.3
	2	5	3	2	60
	3	47	15	32	31.9
	4	1	0	1	0
	6	2	0	2	0
	12	35	10	25	28.6
	15	6	4	2	66.7
	24	13	6	7	46.2
	96	1	0	1	0
**Duration of follow-up (months)**					
	6	31	14	17	45.2
	12	38	13	25	34.2
	24	53	18	35	34
	36	23	14	9	60.9
	60	11	5	6	45.5
	84	5	0	5	0
	240	1	0	1	0
**Phase**					
	1	41	16	25	39
	2	17	6	11	35.3
	3	60	20	40	33.3
	Not applicable	33	15	18	45.5

**Inclusion Criteria**				
Race/Ethnicity	66	26	40	39.4
No standard therapy available, or standard therapy has failed	9	1	8	11
Patients must have received no prior therapy including hormonal therapy, chemotherapy, targeted therapy, immunotherapy or radiation therapy	98	42	5igur6	42.9
None of the above	59	22	37	37.3
**Exclusion Criteria**				
Incarceration	31	14	17	45.2
Pregnancy	48	19	29	39.6
Unable to consent in English	62	23	39	37.1
Evidence of metastatic disease	81	31	50	38.3
Comorbidities	125	51	74	40.8
Previous history of other cancer	102	34	68	33.3
Concomitant medications	13	5	8	38.5
Currently on other chemotherapy regimen	47	15	32	31.9
Previous chemotherapy	44	12	32	27.3
Previous surgery	8	2	6	25

*Screen positive is defined as a patient who was screened positive for clinical trial eligibility.

Table S4: : Multivariable logistic regression for whether patient factors influence trial enrollment overall.

Variable Names	ALL (151)	Patient Enrolled (55)	Patient did not enroll (96)	PVALS	% Enrolled
Insurance status choice Private insurance					
Checked	40 (26.5)	13 (23.6)	27 (28.1)	0.572	32.5
Unchecked	111 (73.5)	42 (76.4)	69 (71.9)		37.8
Insurance status choice Medicare					
Checked	72 (47.7)	22 (40)	50 (52.1)	0.177	30.6
Unchecked	79 (52.3)	33 (60)	46 (47.9)		41.8
Insurance status choice Medicaid					
Checked	29 (19.2)	16 (29.1)	13 (13.5)	0.031	55.2
Unchecked	122 (80.8)	39 (70.9)	83 (86.5)		32
Insurance status choice Free care					
Checked	4 (2.6)	2 (3.6)	2 (2.1)	0.622	50
Unchecked	147 (97.4)	53 (96.4)	94 (97.9)		36.1
Insurance status choice Other					

Checked	7 (4.6)	3 (5.5)	4 (4.2)	0.706	42.9
Unchecked	144 (95.4)	52 (94.5)	92 (95.8)		36.1
Marital Status					
Divorced	14 (9.3)	7 (12.7)	7 (7.3)	0.288	50
Legally Separated	1 (0.7)	0 (0)	1 (1)		0
Married	58 (38.4)	21 (38.2)	37 (38.5)		36.2
Single	61 (40.4)	23 (41.8)	38 (39.6)		37.7
Unknown	3 (2)	2 (3.6)	1 (1)		66.7
Widowed	14 (9.3)	2 (3.6)	12 (12.5)		14.3
Race					
Asian	4 (2.6)	3 (5.5)	1 (1)	0.125	75
Black or African American	72 (47.7)	23 (41.8)	49 (51)		31.9
Unknown/not reported	12 (7.9)	7 (12.7)	5 (5.2)		58.3
White	63 (41.7)	22 (40)	41 (42.7)		34.9
Ethnicity					
Hispanic/LatinX	10 (6.6)	6 (10.9)	4 (4.2)	0.111	60
Non-Hispanic	136 (90.1)	46 (83.6)	90 (93.8)		33.8
Not Available	5 (3.3)	3 (5.5)	2 (2.1)		60
Did this patient have multiple screening events dates					
No	118 (78.1)	47 (85.5)	71 (74)	0.107	39.8
Yes	33 (21.9)	8 (14.5)	25 (26)		24.2
What type of cancer does she have					
Cervical Cancer	23 (15.2)	13 (23.6)	10 (10.4)	0.032	56.5
Endometrial cancer	93 (61.6)	33 (60)	60 (62.5)		35.5
No Cancer (BRCA mut)	3 (2)	0 (0)	3 (3.1)		0
Ovarian cancer	28 (18.5)	7 (12.7)	21 (21.9)		25
Vaginal Cancer	2 (1.3)	2 (3.6)	0 (0)		100
Vulvar Cancer	2 (1.3)	0 (0)	2 (2.1)		0
What stage of cancer does she have					
1	57 (37.7)	24 (43.6)	33 (34.4)	0.087	42.1
2	8 (5.3)	6 (10.9)	2 (2.1)		75
3	34 (22.5)	12 (21.8)	22 (22.9)		35.3
4	38 (25.2)	11 (20)	27 (28.1)		28.9
No cancer	3 (2)	0 (0)	3 (3.1)		0
Unknown	11 (7.3)	2 (3.6)	9 (9.4)		18.2
Histopathologic Subtypes Endometrial choice Endometrioid adenocarcinoma					
Checked	61 (40.4)	22 (40)	39 (40.6)	1	36.1
Unchecked	90 (59.6)	33 (60)	57 (59.4)		36.7
Histopathologic Subtypes Endometrial choice Mucinous carcinoma					

Unchecked	151 (100)	55 (100)	96 (100)	1	36.4
Histopathologic Subtypes Endometrial choice Serous carcinoma					
Checked	21 (13.9)	7 (12.7)	14 (14.6)	0.812	33.3
Unchecked	130 (86.1)	48 (87.3)	82 (85.4)		36.9
Histopathologic Subtypes Endometrial choice Clear cell carcinoma					
Checked	2 (1.3)	1 (1.8)	1 (1)	1	50
Unchecked	149 (98.7)	54 (98.2)	95 (99)		36.2
Histopathologic Subtypes Endometrial choice Squamous cell carcinoma					
Unchecked	151 (100)	55 (100)	96 (100)	1	36.4
Histopathologic Subtypes Endometrial choice Undifferentiated carcinoma					
Unchecked	151 (100)	55 (100)	96 (100)	1	36.4
Histopathologic Subtypes Endometrial choice Mixed carcinoma					
Unchecked	151 (100)	55 (100)	96 (100)	1	36.4
Histopathologic Subtypes Endometrial choice Metastatic carcinoma					
Checked	1 (0.7)	0 (0)	1 (1)	1	0
Unchecked	150 (99.3)	55 (100)	95 (99)		36.7
Histopathologic Subtypes Endometrial choice Carcinosarcoma					
Checked	13 (8.6)	4 (7.3)	9 (9.4)	0.77	30.8
Unchecked	138 (91.4)	51 (92.7)	87 (90.6)		37
Histopathologic Subtypes Cervical choice Squamous carcinomas					
Checked	17 (11.3)	9 (16.4)	8 (8.3)	0.18	52.9
Unchecked	134 (88.7)	46 (83.6)	88 (91.7)		34.3
Histopathologic Subtypes Cervical choice Adenocarcinomas					
Checked	6 (4)	5 (9.1)	1 (1)	0.024	83.3
Unchecked	145 (96)	50 (90.9)	95 (99)		34.5
Histopathologic Subtypes Cervical choice Adenosquamous carcinoma					
Unchecked	151 (100)	55 (100)	96 (100)	1	36.4
Histopathologic Subtypes Cervical choice Mesenchymal tumours sarcomas					
Unchecked	151 (100)	55 (100)	96 (100)	1	36.4
Histopathologic Subtypes Cervical choice Mixed epithelial and mesenchymal tumours					

Unchecked	151 (100)	55 (100)	96 (100)	1	36.4
Histopathologic Subtypes Cervical choice Neuroendocrine carcinoma					
Checked	1 (0.7)	0 (0)	1 (1)	1	0
Unchecked	150 (99.3)	55 (100)	95 (99)		36.7
Histopathologic Subtypes Cervical choice Malignant germ cell tumor					
Unchecked	151 (100)	55 (100)	96 (100)	1	36.4
Histopathologic Subtypes Cervical choice Hematopoietic and lymphoid					
Unchecked	151 (100)	55 (100)	96 (100)	1	36.4
Histopathologic Subtypes Cervical choice Undifferentiated carcinomas					
Unchecked	151 (100)	55 (100)	96 (100)	1	36.4
Histopathologic Subtypes Ovarian choice Serous Cell					
Checked	26 (17.2)	7 (12.7)	19 (19.8)	0.371	26.9
Unchecked	125 (82.8)	48 (87.3)	77 (80.2)		38.4
Histopathologic Subtypes Ovarian choice Germ cell					
Unchecked	151 (100)	55 (100)	96 (100)	1	36.4
Histopathologic Subtypes Ovarian choice Sex cord stromal					
Unchecked	151 (100)	55 (100)	96 (100)	1	36.4
Histopathologic Subtypes Ovarian choice Non specific					
Checked	1 (0.7)	0 (0)	1 (1)	1	0
Unchecked	150 (99.3)	55 (100)	95 (99)		36.7
Histopathologic Subtypes Vulvar choice Squamous cell carcinoma well differentiated not otherwise specified					
Checked	2 (1.3)	0 (0)	2 (2.1)	0.534	0
Unchecked	149 (98.7)	55 (100)	94 (97.9)		36.9
Histopathologic Subtypes Vulvar choice Basaloid carcinoma					
Unchecked	151 (100)	55 (100)	96 (100)	1	36.4
Histopathologic Subtypes Vulvar choice Warty condylomatous carcinoma					
Unchecked	151 (100)	55 (100)	96 (100)	1	36.4
Histopathologic Subtypes Vulvar choice Verrucous carcinoma					
Unchecked	151 (100)	55 (100)	96 (100)	1	36.4

Histopathologic Subtypes Vulvar choice Giant cell squamous carcinoma					
Unchecked	151 (100)	55 (100)	96 (100)	1	36.4
Histopathologic Subtypes Vulvar choice Spindle cell squamous carcinoma					
Unchecked	151 (100)	55 (100)	96 (100)	1	36.4
Histopathologic Subtypes Vulvar choice Acantholytic squamous cell carcinoma adenoid squamous carcinoma					
Unchecked	151 (100)	55 (100)	96 (100)	1	36.4
Histopathologic Subtypes Vulvar choice Lymphoepithelioma like carcinoma					
Unchecked	151 (100)	55 (100)	96 (100)	1	36.4
Histopathologic Subtypes Vulvar choice Basal cell carcinoma					
Unchecked	151 (100)	55 (100)	96 (100)	1	36.4
Histopathologic Subtypes Vulvar choice Metatypical basal cell carcinoma basosquamous carcinoma					
Unchecked	151 (100)	55 (100)	96 (100)	1	36.4
Histopathologic Subtypes Vulvar choice Adenoid basal cell carcinoma					
Unchecked	151 (100)	55 (100)	96 (100)	1	36.4
Histopathologic Subtypes Vulvar choice Sebaceous cell carcinom					
Unchecked	151 (100)	55 (100)	96 (100)	1	36.4
Histopathologic Subtypes Vaginal choice Squamous cell carcinoma					
Checked	2 (1.3)	2 (3.6)	0 (0)	0.131	100
Unchecked	149 (98.7)	53 (96.4)	96 (100)		35.6
Histopathologic Subtypes Vaginal choice Adenocarcinoma					
Unchecked	151 (100)	55 (100)	96 (100)	1	36.4
Histopathologic Subtypes Vaginal choice Sarcoma					
Unchecked	151 (100)	55 (100)	96 (100)	1	36.4
Histopathologic Subtypes Vaginal choice Melanoma					
Unchecked	151 (100)	55 (100)	96 (100)	1	36.4
Histopathologic Subtypes uterine choice Endometrioid adenocarcinoma					
Unchecked	151 (100)	55 (100)	96 (100)	1	36.4

Histopathologic Subtypes uterine choice Adenocarcinoma NOS					
Unchecked	151 (100)	55 (100)	96 (100)	1	36.4
Histopathologic Subtypes uterine choice Adenocarcinoma with squamous differentiation					
Unchecked	151 (100)	55 (100)	96 (100)	1	36.4
Histopathologic Subtypes uterine choice Serous papillary serous					
Unchecked	151 (100)	55 (100)	96 (100)	1	36.4
Histopathologic Subtypes uterine choice Mixed cell adenocarcinoma					
Unchecked	151 (100)	55 (100)	96 (100)	1	36.4
Histopathologic Subtypes uterine choice Clear cell					
Unchecked	151 (100)	55 (100)	96 (100)	1	36.4
Histopathologic Subtypes uterine choice Mucinous adenocarcinoma					
Unchecked	151 (100)	55 (100)	96 (100)	1	36.4
Histopathologic Grading					
High	69 (45.7)	20 (36.4)	49 (51)	0.227	29
Low	38 (25.2)	17 (30.9)	21 (21.9)		44.7
Moderate	25 (16.6)	12 (21.8)	13 (13.5)		48
No cancer	3 (2)	0 (0)	3 (3.1)		0
Unknown	16 (10.6)	6 (10.9)	10 (10.4)		37.5
What is her treatment history prior to enrollment choice Surgery					
Checked	74 (49)	30 (54.5)	44 (45.8)	0.316	40.5
Unchecked	77 (51)	25 (45.5)	52 (54.2)		32.5
What is her treatment history prior to enrollment choice Radiation therapy					
Checked	24 (15.9)	9 (16.4)	15 (15.6)	1	37.5
Unchecked	127 (84.1)	46 (83.6)	81 (84.4)		36.2
What is her treatment history prior to enrollment choice Chemotherapy Immunotherapy					
Checked	65 (43)	25 (45.5)	40 (41.7)	0.733	38.5
Unchecked	86 (57)	30 (54.5)	56 (58.3)		34.9
What is her treatment history prior to enrollment choice Fertility Sparing Treatment					
Checked	2 (1.3)	0 (0)	2 (2.1)	0.534	0
Unchecked	149 (98.7)	55 (100)	94 (97.9)		36.9

What is her treatment history prior to enrollment choice No treatment History					
Checked	49 (32.5)	17 (30.9)	32 (33.3)	0.857	34.7
Unchecked	102 (67.5)	38 (69.1)	64 (66.7)		37.3
Molecular Testing Performed					
No	75 (49.7)	30 (54.5)	45 (46.9)	0.401	40
Yes	76 (50.3)	25 (45.5)	51 (53.1)		32.9
Pertinent Findings from Molecular Testing choice ER					
Checked	26 (17.2)	7 (12.7)	19 (19.8)	0.371	26.9
Unchecked	125 (82.8)	48 (87.3)	77 (80.2)		38.4
Pertinent Findings from Molecular Testing choice PR					
Checked	18 (11.9)	5 (9.1)	13 (13.5)	0.603	27.8
Unchecked	133 (88.1)	50 (90.9)	83 (86.5)		37.6
Pertinent Findings from Molecular Testing choice BRCA1					
Checked	8 (5.3)	4 (7.3)	4 (4.2)	0.463	50
Unchecked	143 (94.7)	51 (92.7)	92 (95.8)		35.7
Pertinent Findings from Molecular Testing choice BRCA2					
Checked	4 (2.6)	1 (1.8)	3 (3.1)	1	25
Unchecked	147 (97.4)	54 (98.2)	93 (96.9)		36.7
Pertinent Findings from Molecular Testing choice MSI					
Checked	8 (5.3)	4 (7.3)	4 (4.2)	0.463	50
Unchecked	143 (94.7)	51 (92.7)	92 (95.8)		35.7
Pertinent Findings from Molecular Testing choice MMR					
Checked	11 (7.3)	7 (12.7)	4 (4.2)	0.099	63.6
Unchecked	140 (92.7)	48 (87.3)	92 (95.8)		34.3
Pertinent Findings from Molecular Testing choice TMB					
Checked	10 (6.6)	5 (9.1)	5 (5.2)	0.498	50
Unchecked	141 (93.4)	50 (90.9)	91 (94.8)		35.5
Pertinent Findings from Molecular Testing choice P53					
Checked	21 (13.9)	6 (10.9)	15 (15.6)	0.474	28.6
Unchecked	130 (86.1)	49 (89.1)	81 (84.4)		37.7
How many screenable events does she have					
1	109 (72.2)	46 (83.6)	63 (65.6)	0.148	42.2
2	34 (22.5)	8 (14.5)	26 (27.1)		23.5
3	6 (4)	1 (1.8)	5 (5.2)		16.7

4	1 (0.7)	0 (0)	1 (1)		0
5	1 (0.7)	0 (0)	1 (1)		0
What trial were they being screened for and found to be screen positive choice SISTER					
Checked	40 (26.5)	12 (21.8)	28 (29.2)	0.346	30
Unchecked	111 (73.5)	43 (78.2)	68 (70.8)		38.7
What trial were they being screened for and found to be screen positive choice Dragonfly					
Checked	7 (4.6)	3 (5.5)	4 (4.2)	0.706	42.9
Unchecked	144 (95.4)	52 (94.5)	92 (95.8)		36.1
What trial were they being screened for and found to be screen positive choice NRG GY018					
Checked	6 (4)	4 (7.3)	2 (2.1)	0.191	66.7
Unchecked	145 (96)	51 (92.7)	94 (97.9)		35.2
What trial were they being screened for and found to be screen positive choice NRG GY020					
Checked	3 (2)	2 (3.6)	1 (1)	0.3	66.7
Unchecked	148 (98)	53 (96.4)	95 (99)		35.8
What trial were they being screened for and found to be screen positive choice CHEMO ID					
Checked	17 (11.3)	2 (3.6)	15 (15.6)	0.031	11.8
Unchecked	134 (88.7)	53 (96.4)	81 (84.4)		39.6
What trial were they being screened for and found to be screen positive choice DIRECT					
Checked	36 (23.8)	12 (21.8)	24 (25)	0.696	33.3
Unchecked	115 (76.2)	43 (78.2)	72 (75)		37.4
What trial were they being screened for and found to be screen positive choice NRG GY019					
Checked	7 (4.6)	4 (7.3)	3 (3.1)	0.258	57.1
Unchecked	144 (95.4)	51 (92.7)	93 (96.9)		35.4
What trial were they being screened for and found to be screen positive choice ROCC					
Checked	12 (7.9)	8 (14.5)	4 (4.2)	0.031	66.7
Unchecked	139 (92.1)	47 (85.5)	92 (95.8)		33.8
What trial were they being screened for and found to be screen positive choice NRG CC010					
Checked	38 (25.2)	11 (20)	27 (28.1)	0.331	28.9

Unchecked	113 (74.8)	44 (80)	69 (71.9)		38.9
What trial were they being screened for and found to be screen positive choice NRG GY026					
Checked	4 (2.6)	1 (1.8)	3 (3.1)	1	25
Unchecked	147 (97.4)	54 (98.2)	93 (96.9)		36.7
What trial were they being screened for and found to be screen positive choice NRG CC008					
Checked	3 (2)	0 (0)	3 (3.1)	0.554	0
Unchecked	148 (98)	55 (100)	93 (96.9)		37.2
What trial were they being screened for and found to be screen positive choice FIERCE					
Checked	8 (5.3)	4 (7.3)	4 (4.2)	0.463	50
Unchecked	143 (94.7)	51 (92.7)	92 (95.8)		35.7
What trial were they being screened for and found to be screen positive choice IO-VANCE					
Checked	1 (0.7)	0 (0)	1 (1)	1	0
Unchecked	150 (99.3)	55 (100)	95 (99)		36.7
What trial were they being screened for and found to be screen positive choice GLORI-OSA					
Checked	5 (3.3)	1 (1.8)	4 (4.2)	0.653	20
Unchecked	146 (96.7)	54 (98.2)	92 (95.8)		37
What trial were they being screened for and found to be screen positive choice ACRIVON					
Checked	4 (2.6)	1 (1.8)	3 (3.1)	1	25
Unchecked	147 (97.4)	54 (98.2)	93 (96.9)		36.7
What trial were they being screened for and found to be screen positive choice STAR					
Checked	1 (0.7)	0 (0)	1 (1)	1	0
Unchecked	150 (99.3)	55 (100)	95 (99)		36.7
Screened for STAR	1 (0.7)	0 (0)	1 (1)	1	0
What trial were they being screened for and found to be screen positive choice A151804					
Checked	1 (0.7)	0 (0)	1 (1)	1	0
Unchecked	150 (99.3)	55 (100)	95 (99)		36.7
What trial were they being screened for and found to be screen positive choice Ad-venchen					
Checked	1 (0.7)	0 (0)	1 (1)	1	0

Unchecked	150 (99.3)	55 (100)	95 (99)		36.7
What trial were they being screened for and found to be screen positive choice NRG GY006					
Unchecked	151 (100)	55 (100)	96 (100)	1	36.4
Comorbidities choice Myocardial infarction					
Checked	8 (5.3)	5 (9.1)	3 (3.1)	0.141	62.5
Unchecked	143 (94.7)	50 (90.9)	93 (96.9)		35
Comorbidities choice Hypertension					
Checked	107 (70.9)	36 (65.5)	71 (74)	0.272	33.6
Unchecked	44 (29.1)	19 (34.5)	25 (26)		43.2
Comorbidities choice Peripheral vascular disease					
Checked	8 (5.3)	2 (3.6)	6 (6.2)	0.711	25
Unchecked	143 (94.7)	53 (96.4)	90 (93.8)		37.1
Comorbidities choice CVA or TIA					
Checked	8 (5.3)	4 (7.3)	4 (4.2)	0.463	50
Unchecked	143 (94.7)	51 (92.7)	92 (95.8)		35.7
Comorbidities choice COPD					
Checked	9 (6)	5 (9.1)	4 (4.2)	0.287	55.6
Unchecked	142 (94)	50 (90.9)	92 (95.8)		35.2
Comorbidities choice Diabetes mellitus					
Checked	44 (29.1)	14 (25.5)	30 (31.2)	0.577	31.8
Unchecked	107 (70.9)	41 (74.5)	66 (68.8)		38.3
Comorbidities choice Moderate to severe CKD					
Checked	21 (13.9)	5 (9.1)	16 (16.7)	0.23	23.8
Unchecked	130 (86.1)	50 (90.9)	80 (83.3)		38.5
Comorbidities choice History of other Cancer					
Checked	19 (12.6)	5 (9.1)	14 (14.6)	0.446	26.3
Unchecked	132 (87.4)	50 (90.9)	82 (85.4)		37.9
Comorbidities choice History of Smoking					
Checked	54 (35.8)	15 (27.3)	39 (40.6)	0.114	27.8
Unchecked	97 (64.2)	40 (72.7)	57 (59.4)		41.2
Comorbidities choice None of the above					
Checked	23 (15.2)	14 (25.5)	9 (9.4)	0.01	60.9
Unchecked	128 (84.8)	41 (74.5)	87 (90.6)		32
ENROLL_GROUP					
Did enroll	55 (36.4)	55 (100)	0 (0)	<.001	100
Medical Factors	32 (21.2)	0 (0)	32 (33.3)		0
Patient Factors	59 (39.1)	0 (0)	59 (61.5)		0

Staffing	5 (3.3)	0 (0)	5 (5.2)		0
YEAR_OF_SCREEN					
2021	2 (1.3)	1 (1.8)	1 (1)	0.254	50
2022	57 (37.7)	25 (45.5)	32 (33.3)		43.9
2023	92 (60.9)	29 (52.7)	63 (65.6)		31.5
Continuous Variables					
Distance.to.UMCNO..miles.	59.27 (137.43)	77.39 (130.5)	48.9 (140.86)	0.032	
Age..in.years.since.screening.	60.67 (13.45)	57.38 (13.35)	62.55 (13.21)	0.021	
How.many.screenable.events.does.she.have.	1.35 (0.66)	1.18 (0.43)	1.45 (0.74)	0.015	
CCI.Score	5.86 (2.85)	5.36 (2.93)	6.15 (2.78)	0.07	
YEAR_OF_SCREEN	2022.6 (0.52)	2022.51 (0.54)	2022.65 (0.5)	0.118	

Disclaimer/Publisher's Note: The statements, opinions and data contained in all publications are solely those of the individual author(s) and contributor(s) and not of MDPI and/or the editor(s). MDPI and/or the editor(s) disclaim responsibility for any injury to people or property resulting from any ideas, methods, instructions or products referred to in the content.
